# The Treatment of Inflammation

**Published:** 1872-05

**Authors:** 


### SELECTIONS

### The Treatment of Inflammation.

Dr. Charles Murchison says in a lecture published in the *British Medical Journal:*

I can here do no more than sketch, in a few lines, some of the leading principles which ought to guide you in the treatment of inflammation.

1. Remove, if possible, the cause of the inflammation. This is the first object always to be aimed at. If it can be effected it will do more good than all other remedies put together. You can not fail to perceive how useless it would be to treat with blood-letting, purgatives, and other measures, an inflammation of the eye, or of the areolar tissue, and depending on the presence of a foreign body as long as the foreign body was allowed to remain. The surgeon has many opportunities of removing the cause of inflammation. He does this, for example, every time that he puts and maintains in opposition the broken ends of a fractured bone, or that he extracts a sequestrum of necrosed bone, or a calculus from the urinary bladder, or that he dilates a stricture of the urethra. The physician, unfortunately, is often unable to say what is the cause of the inflammations which come under his care, or, if he knows the cause, he may be powerless to remove it; still it is in this direction that he ought to strive. When inflammation of the stomach is excited by chemical or other irritants, the first thing to be done is to pump out or neutralize the poison. When inflammation of the same organ is due to the practice of frequent sipping of alchohol, a removal of the cause suffices to effect a cure; and all attempts at cure while the cause remains, are useless. Inflammation of the bowels may be excited by accumulations of feces, and will cease on their removal. In uremic gastro-enteritis the main thing to be done is to endeavor to restore the action of the kidneys; and in inflammaton of the lungs from inhalation of irritating particles of dust, the patient must first of all renounce his injurious occupation. Lastly, in certain cases of protracted pyelitis from the lodgment of a stone in the kidneys, an attempt to remove the stone by surgical means is a feasible operation.

Prevent as far as possible any further stimulus to the inflamed part. Removed light from the inflamed eye and sound from the inflammed ear, and prevent food from entering the inflamed stomach. In gestritis the diet should be restricted to iced milk in small quantities. However low the patient may be, it would be manifestly hurtful to give him alchohol by the mouth; but, if necessary, it may be given by the rectum. For a like reason a person with inflammation of the lungs or kidneys must be preserved from draughts of cold air; and in acute peritonitis from suspected perforation of the bowel, absolute rest of the body is of paramount importance, while the vermicular action of the intestines is suspended by the free administration of opium.

3. Always endeavor to discover the constitutional peculiarities of the individual, and remember that for some varieties of inflammation we possess what may be called specific remedies. Mercury will cure the inflammations of constitutional syphilis, and colchicum with alkalies those of gout. Iodide of postasium seems to act in a specific manner upon periosteal inflammations, chlorate of potash on ulcerative stomatitis, and arsenic on certain inflammations of the skin. Much difference of opinion as you are aware, has existed on the utility of mercury in inflammation. At one time most patients with inflammation were salivated, whereas now the drug is almost universally condemned, not only as useless but as positively injurious. The main cause of the difference of opinion which still exists on the matter is to be found in the nonrecognition of the constitutional varieties of inflammation. In rheumatic pericarditis, mercury has been proved, times without number, to be useless, and in many inflammations to which persons with disease of the kidneys are liable it is always hurtful; but in syphilitic iritis, and syphilitic inflammation of the lungs, liver or other organs, mercury often acts like a charm. I am speaking now of the constitutional effects of mercury when given in frequent small doses. As an occasional aperient, or in conjunction with opium to counteract its stimulating effect, mercury may be useful in other forms of inflammation than the syphilitic.

4. Moderate vascular action in the inflamed part; but, in attempting to do this, keep in view the general condition of the patient. Remembering that increased vascular action is in most instances the chief phenomenon marking the origin and spread of inflammation, it is clear that one of the chief objects in treatment must be to reduce the quantity of blood, and the rapidity of its circulation in the inflamed part. The principal measures which have been recommended for this purpose are as follows:

*a. Blood-letting.*—According to the manner in which the blood is drawn, blood-letting is said to be either *general* or *local.* In the former case, blood is taken from one opening in a large vein, and its removal can only act upon the inflamed part through the general circulation; in the latter case we endeavor by a number of small openings into minute vessels made by leech-bites, or the sacrificator of a cupping instrument to take blood directly from the part which is inflamed. You are aware that blood-letting, both general and local, was at one time the universal treatment of inflammation, but the letting of blood in any way is now one of the rarest of surgical operations. In my introductory lecture I pointed out to you that an attempt had been made to account for this revolution in medical practice on the supposition that inflammations had changed their type; that formerly they were sthenic and required blood-letting, but that now they are asthenic and are injured by depletion, I endeavored to show you, however, that this view of the matter is untenable ; and I need only now repeat that in some parts of the world it is still the fashion to treat inflammations by copious blood-letting, and that it is difficult to imagine how the type of inflammation could have changed in one country and not in another. There can be no doubt that much mischief was done in former days by copious general bleedings in inflammation. In order to diminish effectually the quantity of blood in the inflamed part through the general circulation, it is necessary to take such a quantity of blood that its quality becomes impoverished, while the heart's action is weakened and the reparative powers of the system are impaired. But the same objections does not apply to local bleedings. In many of the inflammations at or near the surface of the body which come under the notice of the surgeon, the effect of local bleeding in relieving pain, diminishing congestion, and otherwise moderating the intensity of the inflammatory process, is so immediate and marked that it is difficult to understand the modern antipathy in this country to blood-letting in any form. It is argued that the loss of even a small quantity of blood weakens the entire system, and especially impairs the vitality of the inflamed part; but such statements have been chiefly advanced by writers who have little or no experience of the effects of blood-letting themselves, and are, as I think, contrary to the evidence of our senses, while repeatedly you will have occasion to observe that a conjestion of the brain or of the lungs is at once relieved by a natural hemorrhage—by a copious epistaxis or hemoptysis. There is one important difference, however, as regards local bleeding, between an inflammation of some internal organ and one on the outer surface of the body. In the latter case there is no difficulty in understanding how local bleeding diminishes the quantity of blood in the inflamed part, but it is not so in the former. When, for instance, you apply leeches to the chest in a case of plurisy, you draw blood from the skin, but how is this to relieve the inflamed pleura? The opponents of blood-letting say that the vessels of the pleura can only be effected through the general circulation, and that the amount of blood drawn by the leeches is much too small to operate beneficially in this way; yet, on calm consideration, you must see that it is not necessary for local depletion to act beneficially that it should do so through the general circulation. It may do so through the nearest arterial trunk, which is in common to the external surface and the inflamed organ. The intercostal artery can only transmit a certain amount of blood, and when the blood is made to flow from its superficial branches, less will go to the deeper branches. But whatever be the explanation there can be no doubt of the clinical fact that the intensity of inflammation may often be moderated by local blood-letting, and this, too, without any injury to the patient. In inflammations, for example, of the liver or intestines, I have repeatedly observed the most marked and immediate relief follow the application of leeches to the abdomen or around the anus. Still you must not have recourse to blood-letting in every case of inflammation. It is only in the early stages of inflammation, or when it is advancing, that you can expect it to do good. You must also abstain from blood-letting in persons of debilitated constitution, or when the inflammation has been excited by an animal poison or some other condition of the blood.

b *Cold.*—The common practice in this country is to apply moist heat to an inflamed part on the surface of the body from the very commencement ; but it does not appear to me that any scientific reason can be given for the practice. Heat increases determination of blood to a part, and therefore seems calculated to increase the inflammation; whereas cold constricts the vessel and diminishes the supply of blood, and in Germany more especially has been found most efficacious in subduing inflammation. On this subject I would advise your reading of an admirable essay by Professor Esmarch of Kiel, entitled *On the Use of Cold in Surgery.* Professor Esmarch declares that of all remedies for surgical inflammations it is most important and that without it he would rather not be a surgeon. The continuous application of cold by means of ice-bags has also been found very efficacious in some inflammations which come under the care of the physician, as in rheumatic inflammation of the joints, and inflammations of internal parts which are near the surface, as the pericardium and peritoneum. But in deep-seated inflammations, cold to the surface may act injuriously by driving blood from the skin to the inflamed part. Cold is also contra-indicated when the inflammatory process is subsiding, and when we have to deal solely with its products. Under the circumstance.

c. *External Heat*, in the form of poultices and fermentations, will be preferable, by means of which we hope to produce a derivation of blood from the part to the skin.

d. *Counter-irritation* by sinapisms, turpentine stupes, and blisters, acts in a similar manner to external heat, and is often of great utility. One caution I would give you respecting blisters: they are often applied in the early stages of inflammation: and when this is near the surface, as in pleurisy, pericarditis, or peritonitis, the irritation may be propagated inward, and increase the mischief which they are intended to allay. It is in chronic inflammations that blisters are most useful.

*e. Purgatives* were at one time used to a mischievous extent in all inflammations; but in inflammations of certain parts of the body they certainly act most efficaciously, by causing a derivation of blood from the inflamed part. In inflammatory affections of the liver, the best treatment is often free purgation. Purgatives in moderation are also useful as one means of elimination from the body the products of inflammatory pyrexia.

*f. Position* may modifiy the amount of blood in any inflamed part. When a limb is inflamed, you take care not to let it hang down; when there is inflammation within the cranium, it is well to keep the head raised; and when there is inflammation of the spinal cord or its membranes, the patient had better not lie on his back.

*g. Belladona*, when applied locally to an inflamed part, causes constriction of the vessels, diminishes the supply of blood, and often gives great relief; but in the case of internal inflammations there is no reason to think that belladonna entering the blood would exert its constricting effect on the vessels in the inflamed in preference to those of any other part.

5. You will treat pyrexia accompaning inflammation according to the principles laid down in my last lecture. You will remember that *aconite* is often of great value for reducing the pulse and temperature in the pyrexia of inflammation, and that *antimony* is especially useful in the same way in inflammations of the mucous membrane of the air passages, where it also appears to relieve conjestion by promoting free transudation to the free surface of the inflamed membrane. As I have already told you, antimony is contraindicated when the heart's action is weak. As regards diet and stimulants, you must be guided by the same rules in inflammation as in pyrexia. In the advanced stages of inflammation, and especially when there is suppuration, ulceration, or gangrene, it will be necessary to give abundance of nutrious food and to stimulate freely. Under these circumstances, also, benefit will be derived from quinine, iron, and the mineral acids.

6. *Sedatives*, particularly *opium* and its preparations, are often of great use in inflammations, for relieving pain, procuring sleep, keeping the inflamed parts at rest, and rendering them less irritable. In acute inflammation of the peritoneum, opium in large and repeated doses is a remedy on which we mainly rely: but opium in any form ought never be given in inflammation of the kidneys, and only with the greatest caution in inflammatory affections of the lungs. In the former case it increases the difficulty which the inflamed kidneys have already to contend with in eliminating the products of pyrexial metamorphosis; in the latter, it leads to the accumulation in the bronchi of inflammatory products, and thus favors a fatal termination by apnœa.

7. Lastly, in the treatment of inflammations you must ever keep in view the dangers likely to arise from derangement of the functions of the inflamed part. In gastritis you will beware of irritating the stomach by drugs or by inappropriate food or stimulants, in extreme cases you will give nutriment for a time entirely by the rectum; in enteritis you will check diarrhea; in the laryngitis you will be permitted to admit air to the lungs by tracheotomy; in accumulations of inflammatory products in the pleura you will consider the propriety of paracentitis; and when the lungs are so extensively inflamed or congested that the portion which remains free does not suffice to transmit the blood of the entire body, and the patient is threatened with asphyxia, you must not scruple in having recourse venesection.

